# Recasting Human Vδ1 Lymphocytes in an Adaptive Role

**DOI:** 10.1016/j.it.2018.03.003

**Published:** 2018-06

**Authors:** Martin S. Davey, Carrie R. Willcox, Alfie T. Baker, Stuart Hunter, Benjamin E. Willcox

**Affiliations:** 1 Cancer Immunology and Immunotherapy Centre, Institute of Immunology and Immunotherapy, School of Medicine and Dental Sciences, University of Birmingham, Birmingham, B15 2TT, UK; 2 Centre for Liver Research and NIHR Biomedical Research Unit in Liver Disease, Institute of Immunology and Immunotherapy, School of Medicine and Dental Sciences, University of Birmingham, Birmingham, B15 2TT, UK; 3 These authors contributed equally

## Abstract

γδ T cells are unconventional lymphocytes commonly described as ‘innate-like’ in function, which can respond in both a T cell receptor (TCR)-independent and also major histocompatibility complex (MHC)-unrestricted TCR-dependent manner. While the relative importance of TCR recognition had remained unclear, recent studies revealed that human Vδ1 T cells display unexpected parallels with adaptive αβ T cells. Vδ1 T cells undergo profound and highly focussed clonal expansion from an initially diverse and private TCR repertoire, most likely in response to specific immune challenges. Concomitantly, they differentiate from a Vδ1 T cell naïve (T_naïve_) to a Vδ1 T cell effector (T_effector_) phenotype, marked by the downregulation of lymphoid homing receptors and upregulation of peripheral homing receptors and effector markers. This suggests that an adaptive paradigm applies to Vδ1 T cells, likely involving TCR-dependent but MHC-unrestricted responses to microbial and non-microbial challenges.

## Highlights

Vδ1 T cells are the predominant tissue-associated γδ T cell subset in humans, and can recognise signs of cellular dysregulation, including viral infection and transformation. They are often assumed to be innate-like effectors.

The Vδ1 TCR repertoire is initially diverse, yet a few clonotypes typically expand heavily over time. Such expanded clonotypes are diverse in sequence both within and between individuals.

Clonal expansion occurs concurrently with differentiation from Vδ1 T_naïve_ to Vδ1 T_effector_ status, alongside a switch from lymphoid to peripheral homing receptors, and upregulation of cytotoxic pathways.

This concurrent expansion and differentiation suggests an adaptive-like response, likely driven by diverse stimuli, including microbial pathogen infection.

## γδ T Cells and the Lymphoid Stress Surveillance Hypothesis

γδ T cells have coevolved alongside αβ T cells and B cells for at least the past ∼450 million years of vertebrate evolution 1, 2, each distinguished by related but distinct somatically recombined antigen receptors. However, our understanding of these different lineages is strikingly imbalanced. Critical to our understanding of αβ T cell and B cells is the classical adaptive paradigm (Box 1). Within this, seminal discoveries have established the core function of the αβ T cell lineage: to enable immune responses to target cells based on the presence on their surface of antigenic peptide in the context of MHC molecules; similarly, we understand that B cells, which underpin humoral immunity, enable the production of soluble antibodies capable of recognising a diverse range of antigenic targets in native, 3D conformation. In keeping with Burnet’s suggestion that ‘receptor occupation’ is key in driving the activation and clonal selection of adaptive lymphocytes [3], structural studies have confirmed both the involvement of clonotypically unique hypervariable loops in αβ TCR/peptide-MHC and B cell receptor (BCR)/antigen engagement, and the significance of such interactions in regulating multiple facets of their immunobiology (Box 1).

Box 1 **Hallmarks of Classical Adaptive Immunity**

Notably, αβ T cells and B cells share key hallmarks of classical adaptive immunity.

**Generation of a Diverse Antigen Receptor Repertoire and Tolerance Mechanisms**

Both αβ T cell and B cell lineages feature somatically recombined TCRs and BCRs, with repertoires featuring high diversity in their hypervariable complementarity-determining region loops, particularly CDR3. For both lineages, selection events during lymphocyte development are critical for immune tolerance. αβ T cells undergo positive and negative selection in the thymus; B cells, in the bone marrow, undergo both antigen-independent positive selection, based on tonic BCR signalling, and processes that eliminate or mitigate autoreactive specificities, including negative selection and anergy induction.

**Clonal Expansion from a Diverse Immune Receptor Repertoire**

The selection of individual clonotypes from within the diverse naïve immune receptor repertoire allows expansion of specific αβ T cell and B cell clonotypes bearing receptors that critically enable amplified responses to specific immune challenges, such as pathogen infection.

**Differentiation into Long-Lived Effectors**

Concurrent with clonal expansion, both αβ T cell and B cell lineages not only undergo differentiation to effectors, but also permit the maintenance of long-lived clonotypically expanded populations, enabling immunological memory, whereby faster and more potent immune responses are induced in response to secondary antigenic challenge.

**Critical Importance of Antigen Receptor–Ligand Interactions**

Diverse studies highlight the central role for TCR–pMHC and BCR–ligand interactions in directing αβ T cell and B cell development, maintenance, clonal amplification and activation, and memory formation, emphatically validating the concept that ‘receptor occupancy’ is a central driver of adaptive lymphocyte biology.

Alt-text: Box 1

Originally identified serendipitously during studies defining αβ TCR genes 4, 5 γδ T cells have by contrast remained somewhat mysterious both in terms of the immunological niche they occupy and the key reason(s) for their evolutionary preservation as a third lymphocyte lineage within vertebrate immunity. Moreover, although γδ T cells are implicated in a range of immune settings, including antimicrobial immunity, antitumour immunity, and tissue homeostasis (reviewed in [6]), the central paradigms that govern their development and antigen recognition functions are unresolved. Finally, despite remaining a focus of ongoing interest, the closely related issue of the importance and exact role of γδ TCR occupation in γδ T cell biology remains a central question.

One concept emerging from mouse studies of γδ T cells is that certain γδ T cell subsets, instead of functioning via conventional adaptive paradigms, may instead act as ‘innate-like’ lymphocytes. Notably, murine γδ T cells express distinct TCRγ and TCRδ combinations at different anatomical sites, and often display semi-invariant TCR repertoires, in some cases featuring highly restricted CDR3 regions 7, 8, 9. They can be preprogrammed during thymic development to differentiate into discrete effector populations producing either interleukin-17 (IL-17) or interferon-gamma (IFN-γ) 10, 11. More recently, intra-epithelial lymphocyte populations have been shown to be selected in tissues after birth, dependent on the expression of particular butyrophilin-like molecules (BTNLs) [12]. Such populations of ‘activated-but-resting’ unconventional lymphocytes are thought to be capable of reacting directly to dysregulated target cells without the need for clonal expansion and differentiation. These data align with the idea such subsets may recognise a limited range of host-encoded stress ligands [13], and suggest that their TCRs act like surrogate pattern recognition receptors (PRRs) for molecular signals of microbial/non-microbial stress. In humans, the γδ T cell subset that aligns most clearly to this biology is characterised by a Vγ9/Vδ2 chain pairing, and represents the predominant peripheral blood subset (1–10% of T cells) [14]. Based on their restricted TCR Vγ and Vδ gene segment usage and CDR3 lengths, presence of common CDR3 motifs, foetal generation, polyclonal production of IFNγ and Tumour Necrosis Factor-α (TNFα) following exposure to pyrophosphate antigens (P-Ags), and strong dependency of TCR-mediated recognition on the BTN3A1 Ig-like protein, this subset arguably conforms to such an innate-like functional paradigm, although the exact mechanisms underlying its recognition of target cells remain unclear. To some extent, the features of these γδ T cell subsets mirror those of unconventional αβ T cell subsets [e.g., mucosal-associated invariant T cells (MAITs) and invariant natural killer cells (iNKTs)], which also feature highly restricted TCR repertoires 15, 16, and have been shown to recognise relatively nonpolymorphic ligands [MHC class I-related gene protein (MR1) and Cluster of differentiation 1 (CD1), respectively].

These observations led to the development of the lymphoid stress surveillance hypothesis [17], which postulates that such effectors, by circumventing the requirement for clonal selection and differentiation, may provide protection from microbial or non-microbial stress challenges during the initial phase of the response, before adaptive immune responses have been generated.

A key finding in this area has been that such subsets can be activated not only via their TCR, but also independently by TCR-extrinsic signals. For example, mouse dendritic epidermal T cells (DETC, a subset of γδ T cells present in murine skin) can be activated directly via NK receptor (NKR)-mediated recognition of stress ligands expressed on stressed epithelium, independently of the TCR [18]. Moreover, while human Vγ9/Vδ2 T cells exhibit potent TCR-dependent recognition of P-Ag-exposed target cells, they can also be activated by NKG2D–ligand interactions and are responsive to cytokines such as IL-12/IL-18 19, 20, 21. In addition, recent studies on mouse skin and gut γδ T cell subsets suggest that TCR signals, potentially mediated via interactions with BTNLs, are required for the development, homing, and establishment of their effector program 11, 12; however, these cells can become hyporesponsive to TCR signals and function in an TCR-independent manner to respond to signs of cellular stress [22].

In addition to Vγ9/Vδ2 T cells, a second human γδ T cell compartment exists, bearing Vδ2-negative TCRs, of which the Vδ1 component is dominant. Vδ1 T cells are the most prevalent subtype of γδ T cells at birth [23], and the dominant γδ T cell subtype in peripheral tissues in adults, such as the gut 24, 25 and skin [26]. Vδ1 T cells have remained very much an enigma in terms of the fundamental paradigms underlying their biology. Based on their predominant effector phenotype, their potent cytotoxicity/cytokine production, combined with their ability to recognise both virally infected and also cancerous cells, and their expression of NKRs 27, 28, 29, they have often been assumed to act in an innate-like fashion, similar to NK cells, potentially enabling recognition of diverse cellular stress signals in target cells. However, here we review recent data that have revised this picture, and suggest instead that Vδ1 T cells exhibit a radical new adaptive immunobiology. These data highlight some of the most significant questions in γδ T cell biology, including the importance and exact role of the γδ TCR that defines the lineage, but ironically, is so poorly understood.

## TCR Repertoire Analyses Reveal Vδ1 T Cell Clonal Amplification

The advent of next-generation sequencing (NGS) approaches has allowed in-depth analyses of the TCR/BCR repertoire within the αβ and B cell lineage, respectively 30, 31. Furthermore, application of these technologies to human peripheral blood γδ T cells has provided valuable information on clonal evolution within the γδ T cell lineage. The approaches used include either DNA-based or RNA-based methods, with the former highlighting the requirement to purify γδ T cells from αβ subsets to avoid contamination of TCRγ sequences recombined in mature αβ T cells [32]. One inherent challenge of these methods is the potential for polymerase chain reaction (PCR) and sequence errors [33]. In the absence of *in silico* error correction and appropriate data handling, this can result in retention of erroneous sequences, often resulting from single-base errors of other higher frequency clonotypes. Therefore, caution should be applied when interpreting highly similar TCR base sequences. The availability of public software packages dedicated to the interpretation of NGS TCR sequencing data (MiTCR [34], MiXCR [35], and TcR [36]), which provide robust error correction, should alleviate such challenges. Moreover, since RNA-based approaches are potentially vulnerable to bias, based on the overall level of RNA differing between cells in different activation and/or differentiation states, ideally parallel techniques, such as single-cell PCR-based TCR analysis, should be considered to validate NGS-based TCR frequencies correlate with cell number on a specific platform 37, 38, 39.

**Figure 1:**
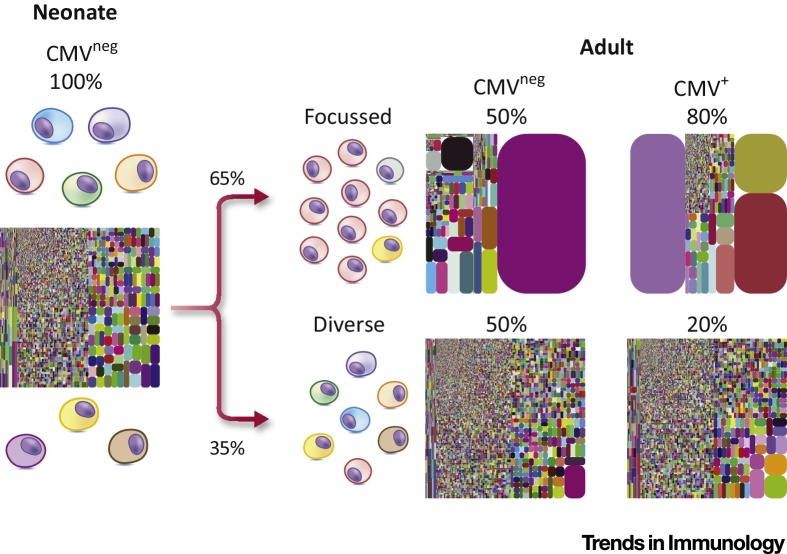
Clonal Expansion in the Vδ1 Repertoire. At birth, neonatal Vδ1 T cell populations comprise a broad set of private clonotypes. During the progression to adulthood, most human Vδ1 T cell repertoires undergo clonotypic focussing towards a limited set of private clonally expanded T cell receptors (TCRs). Despite this, in some individuals, clonal expansion and focussing is not evident and their Vδ1 T cell repertoires remain diverse. While human cytomegalovirus (CMV) infection is directly implicated in driving clonal expansion in Vδ1 T cell repertoires, other immunological stimuli are clearly capable of driving TCR-specific responses. The TCRδ tree plots depict representative Vδ1 T cell repertoires at each stage of life. Each coloured block represents a single unique Complementarity determining region (CDR)-3δ. Each repertoire was private and clonotypes did not overlap between individuals. Percentages are from Davey *et al.*[38], and intended as a guide only.

Such studies have not only shed substantial light on Vδ1 T cell immunobiology, but equally importantly, have also highlighted features of the Vδ1 repertoire that contrast markedly with that of both peripheral blood Vγ9/Vδ2 T cells and thymically programmed mouse γδ T cells. First, based on analyses of cord blood samples, the Vδ1 TCR repertoire at the start of life, which features a variety of Vγ chain pairings, is clonotypically diverse, featuring a range of CDR3 lengths apparently unrelated in sequence. Importantly, these neonatal cord blood Vδ1^+^ repertoires are essentially entirely unfocussed: in other words, it appears that ‘all clonotypes are created equal’, with no single sequence exceeding 1–2% of the repertoire (Figure 1). This contrasts with the Vγ9/Vδ2 population, which, even in foetal and cord blood, includes relatively prevalent Vγ9-JγP clonotypes that are public (i.e., shared at either the nucleotide or amino acid level between individuals) and are present throughout life, consistent with selection of a semi-invariant repertoire preprogrammed in development for polyclonal P-Ag recognition 32, 40, 41, 42.

A second important finding from such studies is that, in comparison to cord blood repertoires, adult Vδ1 repertoires are in general substantially more focussed, typically resulting from the presence of a relatively small number (e.g., <5) of heavily expanded clonotypes, and which often account for a large proportion of the total adult Vδ1 repertoire. Of note, parallel single-cell TCR analysis was used by Davey *et al.* [38] and Ravens *et al.* [39] to confirm that these represented genuine numerical clonal expansions, and were not biased by RNA abundance. Strikingly, both adult and cord blood Vδ1^+^ TCR repertoires were overwhelmingly private (i.e., unique to an individual at both a nucleotide and amino acid level; even more so than TCRβ [38]), with the Complementarity determining region 3 (CDR3) lengths of expanded clonotypes highly diverse, and their sequences apparently unrelated, both within and between individuals [38]. These features appeared to stem from the addition of high levels of nontemplated (N) nucleotides (introduced by terminal deoxynucleotidyl transferase) or occasionally palindromic (P) nucleotides (a mean of 19 N/P nucleotides for Vδ1 CDR3) during variable (diversity) joining [V(D)J] recombination. Both the private nature of the Vδ1 repertoire and the presence of such dominant clonal expansions contrast markedly with the Vγ9/Vδ2 TCR repertoire, which displays less pronounced focussing and contains several public Vγ9 sequences in cord blood and adults 32, 38, 41. Moreover, compared with Vδ1 T cells, the Vγ9/Vδ2 T cell repertoire features restricted CDR3γ and CDR3δ lengths, with CDR3δ2 sequences generally considerably shorter than CDR3δ1 sequences. Similarly, TCRαβ repertoires also display CDR3 length restriction, which is likely imposed by the structural constraints of peptide–MHC recognition [43].

γδ TCR repertoire analyses have also highlighted factors driving such clonal expansions. In particular, Ravens *et al*. showed that acute cytomegalovirus (CMV) infection following stem cell transplantation (SCT) can drive expansion of Vδ2^neg^ (predominantly Vδ1) TCR clonotypes [39]; Vδ repertoires were also noted to be private. These findings build on numerous studies highlighting the importance of Vδ2-negative T cells in responses to CMV infection following kidney transplantation and in healthy donors 29, 44, 45, including spectratyping data consistent with a degree of clonotypic focussing in CMV^pos^ healthy donors [29]. Despite this, the link to CMV infection is not necessarily straightforward, because both Ravens *et al.* [39] and Davey *et al.* [38] noted that the Vδ1 repertoire of some CMV^pos^ individuals lacked clonal expansions, indicating that Vδ1^+^ TCR clonal expansion was not an inevitable consequence of CMV infection (Figure 1). The reason for this is unclear, but could conceivably reflect different routes of infection, greater/lesser dependency of the anti-CMV response on Vδ2^neg^ Vδ1^neg^ γδ T cells, and/or the presence of ‘holes’ in the Vδ1 TCR repertoire in some individuals. Further studies are required to address these possibilities. Moreover, several CMV^neg^ individuals also had heavily expanded Vδ1 clonotypes, indicating that CMV infection is not the sole immune challenge that stimulates Vδ1 T cell responses (Figure 1). Consistent with diverse stimuli for the Vδ1 subset, expansion of Vδ1 T cells has also been noted in response to HIV [46] and in synovial fluid in Lyme disease [47], while two case reports describe Vδ1 clonal focussing after EBV infection in SCT 48, 49. Nevertheless, infectious stimuli underlying clonotypic focussing in healthy donors, other than CMV, remain to be identified. Finally, irrespective of the stimuli inducing such responses, both Davey *et al.* [38] and Ravens *et al.* [39] provide evidence that such expanded clonotypes can be long-lived, and persist for at least 2 years, consistent with long-term contributions to immunosurveillance.

## Clonal Selection in Vδ1 T Cells Induces Adaptive Changes in Phenotype and Function

**Figure 2:**
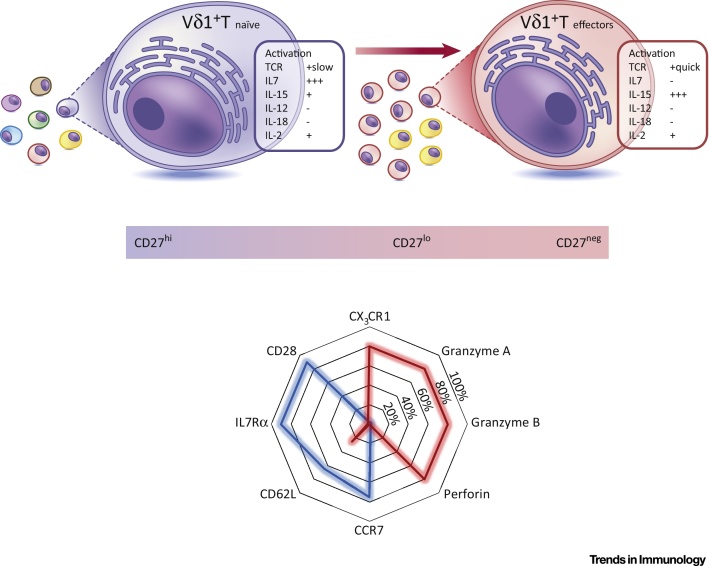
Phenotypic Changes in Vδ1 T Cells upon Adaptive Expansion. Vδ1 T cells displaying a diverse T cell receptor (TCR) repertoire expressed high levels of Cluster of differentiation 27 (CD27). Conversely, clonally focussed TCR repertoires either displayed reduced expression or had completely downregulated CD27. These CD27^hi^ and CD27^lo/neg^ Vδ1 T cells also displayed markers and functional responses consistent with naïve and effector T cells, respectively. Vδ1 T_naïve_ cells, alongside a broad γδ TCR repertoire, expressed the co-stimulatory receptor CD28, lymphoid tissue homing receptor CCR7, tissue access molecule CD62L, and mounted a proliferative response to the lymphoid tissue-associated homeostatic cytokine interleukin 7 (IL-7). By contrast, Vδ1 T_effector_ cells had downregulated CD28, CCR7, CD62L, and IL7Rα and upregulated cytotoxic granzymes, perforin, and endothelial homing receptor CX3C chemokine receptor 1 (CX_3_CR1), and proliferated in response to the peripheral tissue-associated cytokine IL-15. Both populations were unresponsive to innate stimuli (IL-12 and IL-18) but retained TCR responsiveness (anti-CD3 stimulation), with Vδ1 T_effector_ cells becoming rapidly activated, whereas Vδ1 T_naïve_ cells responded over a longer period of time.

Davey *et al.* [38] combined NGS TCR sequencing, single-cell TCR analyses, and a flow cytometric immunophenotyping approach to delineate different Vδ1 subsets in adult peripheral blood and cord blood samples. Notably, a naïve-like CD27^hi^ Vδ1 subset was identified expressing highly diverse TCRs and multiple markers common to naïve T cells, including IL-7R, CD28, CD62L, and CCR7 (Figure 2); we hereafter apply the term ‘Vδ1 T_naïve_’ to this subset. Importantly, although such T_naïve_ cells were typically a minor fraction of adult peripheral blood Vδ1 T cells, essentially the entire Vδ1 T cell subset in cord blood was clonotypically unfocussed. By contrast, clonotypically expanded Vδ1 TCRs present in adults invariably resided within a differentiated effector CD27^lo/neg^ compartment largely absent in cord blood, which was detected to different extents within adult peripheral blood Vδ1 T cells across a 20-person cohort. This compartment shared several phenotypic features with conventional Teffector populations, including expression of granzymes, perforin, and CX3C chemokine receptor 1 (CX_3_CR1; Figure 2); we hereafter apply the term ‘Vδ1 T_effector_’ to this subset. Importantly, the observation that the Vδ1 compartment of the minority of adult donors who retained relatively diverse Vδ1 TCR repertoires was dominated by Vδ1 T_naïve_ cells confirmed the validity of this phenotypic distinction, and highlighted that clonal expansion and differentiation were not inevitable consequences of Vδ1 T cell maturation.

**Figure 3:**
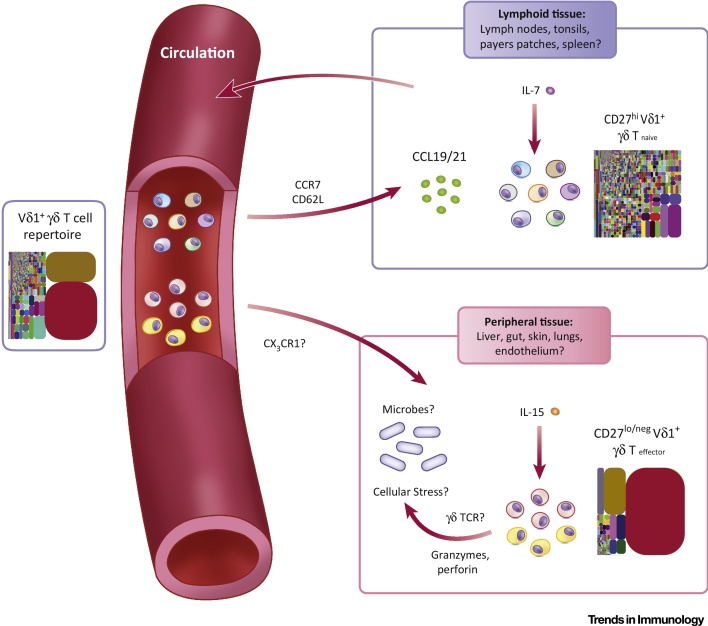
Adaptive Stress Surveillance Paradigm. Both Vδ1 T_naïve_ and T_effector_ cells circulate in the peripheral blood. Vδ1 T_naïve_ populations (expressing CCR7 and CD62L) are likely to migrate to secondary lymphoid tissue, via CCL19 and CCL21 chemokine gradients. Access to secondary lymphoid tissue permits encounter of homeostatic interleukin 7 (IL-7), maintaining Vδ1 T_naïve_ cells and allowing their persistence throughout adulthood. Vδ1 T_naïve_ cells may also encounter cognate antigen either in the lymphoid tissues, akin to αβ T cells, or elsewhere, and give rise to Vδ1 T_effector_ cells. Circulating Vδ1 T_effector_ populations may enter peripheral tissues, accessing homeostatic IL-15 concentrations. Access to peripheral tissues may indicate a stress surveillance role and antimicrobial function, through T cell receptor (TCR)–ligand engagement.

These phenotypic features of Vδ1 T_naïve_ and T_effector_ subsets point towards an adaptive biology. First, the transition from Vδ1 T_naïve_ to T_effector_ subset is accompanied by a reprogramming of homing receptor expression. Vδ1 T_naïve_ cells uniformly express high levels of central lymphoid homing markers. By contrast, Vδ1 T_effector_ cells exhibit strong downregulation of CCR7 and CD62L, but increased CX_3_CR1, which binds to fractalkine, an endothelial homing chemokine. The respective expression profiles of these markers on Vδ1 T_naïve_ and T_effector_ subsets closely mirrored expression on CD8 naïve and T_EMRA_ cell populations, respectively. Correspondingly, and as suggested for CX_3_CR1^hi^ CD8 memory T_effector_ cells [50], CX_3_CR1^hi^ Vδ1 T_effector_ cells may be involved in endothelial immunosurveillance. In terms of function, whereas Vδ1 T_naïve_ cells, such as naïve CD8 T cells, were devoid of cytotoxic effector markers (e.g., perforin, and granzymes A and B), these were heavily upregulated in Vδ1 T_effector_ cells (equivalent to CD8 T_EMRA_ populations). Moreover, Vδ1 T_effector_ cells retained a rapid proliferative capacity and TCR sensitivity, and were preferentially sensitive to IL-15 relative to Vδ1 T_naïve_ cells, which conversely (and similar to naïve CD8 T cells) were preferentially responsive to IL-7 and exhibited slower proliferation following TCR stimulation. These findings strongly suggest that the TCR-diverse CD27^hi^ and highly TCR focussed CD27^lo/neg^ populations represent *bona fide* naïve and effector Vδ1 subsets, respectively (Figures 2 and 3).

These findings demonstrate that, far from representing a preformed effector subset from birth, Vδ1 T cells are initially highly naïve in phenotype and feature an entirely unfocussed TCR repertoire. They also suggest that differentiation to an effector phenotype is not an inevitable developmental process, but is inextricably linked to clonal amplification, and drastically affects both cytotoxic capability and homing receptor expression (Figure 3).

## Adaptive MHC-Unrestricted γδ T Cell Stress Surveillance: A Paradigm for Vδ1 T Cells

The observations outlined above highlight surprising parallels between Vδ1 T cells and classical adaptive T cell subsets, particularly CD8 T cells; conversely, they emphasise key distinctions between Vδ1 T cells and the Vγ9/Vδ2 T cell subset. They suggest that the γδ TCR is central to the biology of Vδ1 T cells, and lead us to suggest a previously unrecognised mode of MHC-unrestricted adaptive immunobiology applies to the Vδ1 T cell subset (Box 2). This paradigm and the evidence that underpins it, has several implications worthy of consideration that will likely frame future investigations.

Box 2 **Key Principles of Adaptive Stress Surveillance**

We propose that three key tenets of an adaptive immunobiology apply to Vδ1 T cells. We suggest that:

(i) specific stress stimuli, including microbial infection and/or colonisation, are capable of triggering the clonal expansion and differentiation of Vδ1 T_effector_ clonotypes from a highly diverse, unfocussed Vδ1 T_naïve_ pool;

(ii) as for αβ T cells and B cells, ‘receptor occupancy’, in this context the ability of the Vδ1 γδ TCR to engage cognate ligands, drives clonal expansion and initiates differentiation to the effector state; and

(iii) clonally expanded Vδ1 T_effector_ populations, which are relatively long-lived, provide enhanced protection against recurrent immune challenges.

Alt-text: Box 2

### Tolerance Induction

The Vδ1 and Vγ9/Vδ2 T cell subsets develop at different stages, consistent with a distinct underlying immunobiology and TCR repertoire for the two compartments. Vγ9/Vδ2 T cells are generated during development in the foetal liver, and later the foetal thymus, and are only present in small numbers in the postnatal thymus 51, 52. Development may require positive selection for BTN3A1 reactivity and/or self phosphoantigens, such as isopentenyl pyrophosphate (IPP), and subsequent expansion after microbial exposure during early childhood [53]. By contrast, Vδ1 T cells are the dominant γδ T cell population in the postnatal thymus. It is unclear whether human γδ T cells undergo thymic selection. The diversity of CDR3 lengths and sequences for Vδ1 and associated Vγ TCR chains would suggest a strong potential for autoreactivity; however, there is little evidence of negative selection in γδ T cells [32]. Alternatively, peripheral tolerance mechanisms may be involved in deleting or inducing the anergy of strongly autoreactive cells, or other mechanisms similar to NK licensing or arming (reviewed in [54]) may be used to set thresholds for Vδ1 reactivity. Vδ2^neg^ γδ T cells express inhibitory NKRs, such as LILRB1/ILT2, which may be involved in this process [28]. Additionally, TCR self-reactivity may involve low-affinity TCR–ligand interactions and may require additional co-stimulatory signals or adhesion molecules to lead to productive TCR signalling [55].

### T Cell Priming and Migration

Although the cellular mechanisms underlying initiation of Vδ1 clonal expansions are unclear, the immune phenotype of Vδ1 T_naïve_ cells suggests preferential access to secondary lymphoid organs, as opposed to clonally expanded Vδ1 T_effector_ subsets. This implies that Vδ1 T_naïve_ cells most likely recirculate between blood and lymph tissues (Figure 3), and suggests that clonal amplification requires lymphoid tissue-derived factors and/or is initiated during a priming step in secondary (or possibly tertiary) lymphoid organs. Future studies assessing the differentiation status and localisation of Vδ1 T cells in secondary and/or tertiary lymphoid organs will be required to address these suggestions, and may shed light on whether specific antigen-presenting cell types are involved in priming. Conversely, the increased expression of CX_3_CR1 on Vδ1 T_effector_ populations and their enhanced sensitivity to IL-15 suggests that they preferentially home to solid tissues, and contribute to a more clonotypically focussed repertoire at such sites. Further studies comparing the phenotypic features and TCR diversity of peripheral blood Vδ1 T cells with those in solid tissues will address these issues.

### Mechanisms and Targets Underpinning Vδ1 γδ TCR Ligand Recognition

Defining the ligands recognised by the γδ TCR remains a major goal and one that may help unlock the molecular basis by which γδ T cells recognise abnormal target cells (Table 1).

**Table 1:** γδ TCR Ligands in the Context of Adaptive Stress Surveillance^a^

Candidate γδ TCR ligand	Chain usage of T cells	Origin of T cells	Frequency of response	Memory phenotype of T cells	Direct ligand binding/affinity	CDR3 involvement	Comments/potential physiological significance	Refs
EPCR	Vγ4/Vδ5	PBMC from immunosuppressed lung transplant patient with acute CMV	25% of total CD3^+^ T cells in one CMV^+^ individual	CD45RO^neg^ CD28^neg^	∼90 μM (BIAcore)	Yes; CDR3γ, CDR3δ^b^	Single private clonotype Expanded clonotype, likely γδ T_effector_ status Potential ‘restriction factor’ for endothelial cells during CMV infection Allows detection of ‘multimolecular stress signature’ on CMV-infected cells EPCR upregulated on various cancer cells	27, 28, 55, 65, 66
PE	Vδ1^+^, various Vγ chains	PE staining of healthy donor blood	0.025% of total CD3^+^ T cells	ND	Mouse TCR-PE 2.7 μM (BIAcore) Human TCR − ND	Yes (mouse γδ TCR)	Various clonotypes involved Collectively low frequency in Vδ1 T cells; therefore, unlikely to represent dominant γδ T_effector_ expansions PE derived from marine blue-green algae; therefore, may reflect potential of Vδ1^+^ T cells to recognise foreign antigens Immunisation of mice upregulated activation markers on PE^+^ γδ T cells and led to IL-17 production	[62]
CD1d	Vδ1^+^, various Vγ chains	CD1d (unloaded or with various lipids) tetramer staining of healthy donor PBMC	<0.05% of total T cells	ND	16 μM α-GalCer/CD1d 35 μM unloaded CD1d (BIAcore); 33 μM sulfatide/CD1d, 240 μM unloaded CD1d (BLI)	CDR3δ required	Various clonotypes involved Collectively low frequency in Vδ1 T cells; therefore, unlikely to represent dominant γδT_effector_ expansions May reflect γδT_naïve_ population CD1d-reactive γδ T cells could expand upon recognition of CD1d-restricted stress and/or infection-linked lipids	61, 63, 64
CD1c	Vδ1^+^, various Vγ chains	CD1c (loaded with Mtb or self lipids) tetramer staining of healthy donor PBMC	0.16% of total T cells	ND	23–30 μM foreign lipids; 28–150 μM self lipids LPA, LPC, sulfatide (BLI)	Yes: chain swap	Various clonotypes involved Collectively low frequency in Vδ1 T cells; therefore, unlikely to represent dominant γδ T_effector_ expansions May reflect γδ T_naïve_ population CD1c-reactive γδ T cells could expand upon recognition of CD1d-restricted stress and/or infection-linked lipids	[60]
Annexin A2	Vδ3^+^ clone	Healthy donor PBMC cultured with Raji + IL-2	ND	ND	3 μM (BIAcore)	ND	Various clonotypes involved Frequency of clone unclear in original donor; may reflect γδT_naïve_ population Annexin A2 upregulated in cellular stress (tumourigenesis, oxidative stress) May permit Vδ2^neg^ γδ T cells to recognise tumour cells or metabolically stressed cells	[59]

a Abbreviations: CCR7, C-C chemokine receptor type 7; EBV, Epstein–Barr virus; EPCR, endothelial protein C receptor; ICAM-1, intercellular adhesion molecule 1; LILRB1/ILT2, leukocyte immunoglobulin-like receptor B1/immunoglobulin-like transcript2; ND, not determined; NKG2D, natural killer group 2 member D; PBMC, peripheral blood mononuclear cell; Skint, selection and upkeep of intraepithelial T cells.

b Carrie Willcox, unpublished data.

Most γδ TCR ligand investigations have focussed on peripheral blood populations. Importantly, our understanding of the Vδ1 TCR repertoire in solid tissues is limited. However, a recent study highlighted a Vγ4Vδ1 subpopulation, present at variable frequencies in colorectal tissue in different individuals, which underwent TCR-dependent activation in response to BTNL3/8-positive target cells [12]. A priority for future investigations is to understand whether BTNL3/8 act as direct ligands for this Vγ4Vδ1 subpopulation, and whether the TCR repertoire and phenotype of such cells reflect a semi-invariant innate-like paradigm or an adaptive immunobiology. In addition, a more comprehensive understanding of the Vδ1 TCR repertoire in different solid tissues is required.

Current data on peripheral blood Vδ1 T cells indicate a highly diverse TCR repertoire both in terms of Vγ chain pairing and Vδ1/Vγ CDR3 regions. Notably, there are no obvious similarities in the CDR3 regions of clonally amplified Vδ1 TCRs, either in terms of lengths or sequences. Moreover, these features may apply to other Vδ2-negative TCRs [38]. The high degree of CDR3 diversity within the Vδ1 TCR repertoire, including use of diverse Vγ gene segments, contrasts markedly with other unconventional T cell populations thought to recognise single germline-encoded ligands (e.g., iNKTs [15], MAITs [16], and Vγ9/Vδ2 T cells 38, 39), which feature semi-invariant TCR repertoires, including prominent public CDR3 clonotypes; by contrast, the Vδ1 TCR repertoire is both diverse and essentially private 38, 39.

Importantly, the high diversity of the peripheral blood Vδ1 TCR repertoire, including in expanded clonotypes, may not necessarily exclude recognition of a limited range of physiologically relevant ligands shared between individuals. Of note, there is precedent for degeneracy in αβ TCR recognition, whereby the same peptide–MHC complex can be recognised by TCRs of diverse sequence [56]. Similarly, antibodies featuring diverse CDR loops can recognise the same protein antigen, either via distinct or similar surface epitopes 57, 58. However, an alternative, and possibly more likely scenario, is that the high Vδ1 TCR diversity reflects a diversity of ligands recognised. This is arguably supported by the diverse array of ligands proposed for Vδ2-negative TCRs 55, 59, 60, 61, 62 (Table 1). A major challenge is to make sense of this seemingly unrelated group of ligands and decipher key underlying principles; however, the basis for making such judgements has hitherto remained entirely unclear. Based on the central assumption that TCR–ligand binding (‘receptor occupation’) drives clonal expansion, the adaptive paradigm we outline suggests a set of criteria (based on clonotype frequency, phenotype, CDR3 involvement, and ligand expression pattern; Table 1) by which the position of candidate ligands within this adaptive framework could be assessed.

Re-evaluation of current γδ T cell ligands in light of these criteria prompts several observations. Since reactivities to CD1c [60] and CD1d 61, 63, 64 described to date reflected extremely low percentages of the Vδ1 T cell repertoire (contributing <0.05% and <0.16% of total T cells, respectively; Table 1), they are unlikely to represent *in vivo* expanded Vδ1 T_effector_ clonotypes. However, they could derive from Vδ1 T_naïve_ subsets, and conceivably changes in lipid cargo in different physiological settings may drive TCR-mediated Vδ1 clonal expansions. Similarly, phycoerythrin (PE) [62], a model BCR antigen derived from a marine alga, was also convincingly demonstrated to be a direct γδ TCR ligand in mice, and for an extremely low proportion of the human Vδ1 T cell repertoire (<0.025% of CD3^+^ T cells, Table 1), excluding recognition by expanded Vδ1 T_effector_ clonotypes. Despite this, PE recognition may reflect the potential of Vδ1 TCRs to recognise foreign antigens in intact form.

One human γδ TCR reactivity, to Endothelial Protein C Receptor (EPCR) [55], arguably fulfils the criteria for recognition by an expanded Vδ1 T_effector_ clonotype, albeit involving a Vδ5 TCR (Table 1). In addition, the LES TCR that recognised EPCR was a private TCR sequence, in keeping with the properties of Vδ1 TCRs and the finding that recognition of EPCR was restricted to a single patient. Despite this, it enabled γδ TCR and EPCR-mediated recognition of CMV-infected fibroblast and/or endothelial cells. Interestingly, although EPCR expression was not enhanced by CMV infection, LES γδ T cell activation was dependent on a TCR-extrinsic ‘multimolecular stress signature’, which included induction of increased expression of intercellular adhesion molecule 1 (ICAM-1) on target cells after CMV infection [55]. Moreover, while similar changes were evident in some tumour lines, it is also clear that overexpression of EPCR itself is linked to genetic changes during tumourigenesis [65], and has also been linked to chemoresistance [66]. Conceivably, the dependence of effector function on TCR-extrinsic changes in addition to the presence of TCR ligands could be an important factor in the maintenance of γδ T cell tolerance in the absence of relevant microbial and/or non-microbial stress stimuli. If, as previously suggested, the LES–EPCR reactivity proves to be ‘unique but paradigmatic’, then other private ligands may map onto other private expanded clonotypes. A repertoire-based ligand identification strategy, ideally focussing on multiple γδ TCR specificities expanded in response to a single immune challenge, should confirm this, and may reveal commonalities between ligands (e.g., expression on a particular tissue relevant to the specific pathogen infection). In addition, other modes of stress stimulus-induced γδ TCR–ligand-mediated activation could be envisaged, for example involving increased expression of the ligand, altered post-translational modification of the ligand, or decreased levels of target cell-expressed ligands for γδ T cell inhibitory receptors. Clearly, future studies in this area, guided by the criteria outlined above, may resolve some of these key questions.

### Nature of Immune Stimuli for Vδ1 Responses

Although CMV appears to trigger clonal expansion of Vδ1 T cells, the fact that CMV-seronegative adults still frequently harbour major Vδ1 T_effector_ populations indicates that other immune stimuli, possibly other infectious challenges, must trigger specific Vδ1 responses. Consistent with this, several pathogens have been linked with increased numbers or clonality of Vδ2-negative T cells, including HIV [46], Epstein–Barr virus (EBV) 48, 49, and other microbial infections [1]. Despite this, the link between a given pathogen infection, specific Vδ1 clonal expansion, and an augmented recall response to that challenge warrants further study. Several reasons suggest that the transition from Vδ1 T_naïve_ to T_effector_ status accompanying such clonal expansions would increase the speed and potency of effector responses: notably, Vδ1 T_effector_ cells express perforin and granzymes, whereas Vδ1 T_naïve_ cells do not; moreover, Vδ1 T_effector_ cells exhibit enhanced and more rapid production of cytokines and proliferation relative to Vδ1 T_naïve_ cells following CD3/CD28 stimulation [38]. In addition, previous studies have highlighted Vδ2-negative T_effector_ responses to CMV-infected target cells following CMV infection [27], alongside an increased proportion of Vδ1 T cells bearing an effector phenotype relative to CMV-seronegative individuals [29]. However, an increased understanding of how such alterations link with clonotypic changes is needed. Given the diverse stimuli that could underpin the generation of clonotypic Vδ1 T_effector_ responses, this will require analysis of human samples before and after relevant infections, and comparison with individuals who either remained uninfected or did not exhibit postinfection clonal expansions. Such analyses will also allow the relative kinetics of the phenotypic transitions and clonal expansion to be assessed.

### Evolutionary Advantage of Adaptive MHC-Unrestricted γδ T Cell Stress Surveillance

The universal presence of γδ T cells in vertebrates suggests that compelling reasons must exist for their evolutionary conservation [1]. In addition to providing semi-invariant γδ T cell populations that have evolved recognition modes highly distinct from αβ T cell subsets, potentially involving germline-encoded targets, such as BTN3A1 67, 68 and BTNL/Selection and upkeep of intraepithelial T cells (Skint) family members 12, 69, it is interesting to consider what evolutionarily advantageous immune functions adaptive γδ T cell subsets might alternatively provide. With regards to CMV infection, the only pathogenic challenge currently confirmed as driving the clonotypic expansion of human Vδ1 T cells, much evidence supports a role for NK cells [70] and CD8 T cells [71] in anti-CMV immunity. However, CMV has also evolved numerous immune evasion strategies targeted at disrupting essential components of CD8/NK immunosurveillance, including the MHC presentation pathway [72], and sequestration of ligands for conserved activatory ligands for germline-encoded NKRs, such as natural killer group 2 member D (NKG2D) [70]. Evolution of a stochastically recombined immune receptor repertoire allowing MHC-unrestricted recognition of ‘altered/stressed self’ via diverse and potentially private γδ TCR reactivities to intact cell surface antigens, would likely complement such strategies and may prove more challenging for pathogens to evade. Conceivably, such subsets could also enable direct recognition of foreign pathogen proteins on the surface of infected host cells. Recent data from mouse models suggesting that γδ T cells provide as potent protection against CMV as the CD8 T cell compartment 73, 74 are consistent with these ideas; however, further studies are required to understand the immunobiology of the γδ subsets involved and the molecular basis of the recognition events in which they are involved.

## Concluding Remarks

Recent studies have substantially revised our understanding of Vδ1 T cells. The coincident clonal expansion and differentiation of Vδ1 T cells not only represent a ‘smoking gun’ that the γδ TCR is likely to be central to their immunobiology, but also lead us to propose that Vδ1 function is underpinned by a novel MHC-unrestricted adaptive paradigm. This contrasts with Vγ9/Vδ2 T cell immunobiology, which appears to be predominantly innate-like, highlighting the coexistence of adaptive and innate-like paradigms within the human γδ T cell lineage.

This adaptive perspective for Vδ1 T cells prompts a reassessment of previous studies, and also provides an intellectual framework around which future investigations can be designed, which should help answer the many unresolved questions (see Outstanding Questions). Of these, the question of what represent *bona fide* physiological ligands for Vδ1 γδ TCRs in the context of this adaptive paradigm is central, but importantly the repertoire and immunophenotype-based observations upon which the paradigm is built suggest objective criteria by which to assess such reactivities and plan future studies. Moreover, there is an expanding evidence base for the importance of Skint/BTN/BTNL molecules in mouse γδ T cell development and biology 12, 69, 75. Understanding the full significance of this family in human γδ T cell biology in the context of parallel innate-like and adaptive-like paradigms is another aim.

An improved understanding of human γδ T cell biology will hopefully accelerate therapeutic exploitation of their function. Despite limited understanding of γδ TCR ligand recognition, there is already substantial therapeutic interest in γδ T cells, particularly in the cancer immunotherapy arena, not least due to their MHC-unrestricted recognition of target cells and potent cytotoxic function. In addition, the ability to immunophenotypically delineate Vδ1 T_naïve_ from T_effector_ subpopulations should provide a means to probe Vδ1 T cell responses against diverse microbial and non-microbial immunological challenges at different life stages, which would represent an important early step along the pathway towards the successful therapeutic exploitation of γδ T cell function.

Outstanding Questions What thymic processes shape the Vδ1 repertoire?

How is Vδ1 clonal amplification initiated? Does this occur in lymphoid organs or in peripheral tissues and, if so, which factors and/or antigen-presenting cells are involved?

When do adaptive Vδ1 responses occur throughout life? Do they compensate for when αβ T cell responses are suppressed, such as in neonates or after transplantation?

Aside from CMV, which stress stimuli trigger clonal selection? What is the range of pathogens involved, and which non-microbial stimuli can drive Vδ1 responses *in vivo*?

What is special about clonally expanded Vδ1 TCRs? Are they capable of recognising ligands that relate to physiologically relevant stress stimuli and, if so, what are these? Are the ligands private to each individual or public, and how do they relate to each other?

Why are the Vδ1 repertoires of some individuals more clonotypically focussed than others? Does this reflect differences in pathogen exposure, or are there ‘holes’ in the Vδ1 repertoires of some individuals with respect to specific stress challenges?

How do the repertoire and immunobiology of Vδ1 T cells in solid tissues relate to peripheral blood Vδ1 T cells? Do they overlap with adaptive Vδ1 subsets in peripheral blood, or are they distinct, and might they also contain semi-invariant populations, as defined in mouse tissue-associated γδ T cells?

Is there a role for BTNL molecules (e.g., BTNL3/8) in the development, selection, or antigenic stimulation of the Vδ1 T cell compartment? If so, does this involve innate-like or adaptive Vδ1 populations?

How phenotypically plastic are Vδ1 T cells? Can they be exploited therapeutically, such as in vaccination or adoptive transfer approaches?
